# Use of extracorporeal membrane oxygenation in children with burn injury: Case report and literature review

**DOI:** 10.1097/MD.0000000000034029

**Published:** 2023-06-16

**Authors:** Yanfei Wang, Kelei Deng, Junjie Qian, Linhua Tan

**Affiliations:** a Department of Surgical intensive care, Children’s Hospital, Zhejiang University School of Medicine, National Clinical Research Center for Child Health, Hangzhou, Zhejiang, China; b Department of Surgical intensive care, Children’s Hospital, Zhejiang University School of Medicine, National Clinical Research Center for Child Health, Hangzhou, Zhejiang, China; c Department of Surgical intensive care, Children’s Hospital, Zhejiang University School of Medicine, National Clinical Research Center for Child Health, Hangzhou, Zhejiang, China; d Department of Surgical intensive care, Children’s Hospital, Zhejiang University School of Medicine, National Clinical Research Center for Child Health, Hangzhou, Zhejiang, China.

**Keywords:** burn encephalopathy, burns, case report, ECMO, inhalation injury

## Abstract

Burns are one of the most debilitating injuries in the world and one of the major causes of accidental disability and death among children. Severe burns can result in irreversible brain damage, placing patients at high risk of brain failure and high mortality. Therefore, timely diagnosis and treatment of burn encephalopathy are crucial for improving prognosis. In recent years, extracorporeal membrane oxygenation (ECMO) has been increasingly used to improve the prognosis of patients with burns. Here, we report a case of ECMO treatment in a child with burns and review the relevant literature.

**Patient concerns:**

A 7-year-old boy with a modified Baux score of 24 presented with asphyxia, loss of consciousness, refractory hypoxemia, and malignant arrhythmia after smoke inhalation for 1 day. Fiberoptic bronchoscopy revealed a large amount of black carbon-like substances aspirated from the trachea.

**Diagnoses:**

Considering that the boy inhaled a large amount of smoke, the clinical manifestation was unclear consciousness, laboratory examination revealed continuous low blood oxygen saturation, and bronchoscopy revealed a large amount of black carbon-like substances in the trachea, thereby leading to the diagnosis of asphyxia, inhalation pneumonia, burn encephalopathy, multiple organ dysfunction syndrome, and malignant arrhythmia. In addition, pulmonary edema and carbon monoxide poisoning are caused by chemical agents, gas fumes, and vapors.

**Interventions:**

The boy’s blood oxygen saturation and blood circulation remained unstable despite various ventilation methods and medications, thus we decided to use ECMO. After 8 days of ECMO support, the patient was successfully weaned from the machine.

**Outcomes:**

Under the application of ECMO, the respiratory and circulatory systems significantly improved. Nevertheless, due to the progressive brain injury caused by burns and the poor prognosis, the parents ceased all treatment and the boy passed away.

**Lessons:**

This case report demonstrates that brain edema and herniation can arise as phenotypes of burn encephalopathy, which is a challenge to treat in children. Children with confirmed or suspected burn encephalopathy should undergo diagnostic tests completed as soon as possible to confirm the diagnosis. After receiving ECMO treatment, the respiratory and circulatory systems of the burn victims reported significantly improved. Hence, ECMO is a viable alternative for supporting patients with burns.

## 1. Introduction

Burns are tissue damage caused by heat, and they have a high incidence, disability, and mortality rate. It is one of the most traumatic events that cause physical and psychological damage to children and imposes significant burdens on families and society as a whole. According to the World Health Organization, 11 million cases of various types of burns occur annually worldwide, and 180,000 people perish as a result. In 2019, there were 8378,122 new cases of burns reported globally. The majority of new cases were centered in the 10 to 19-year-old age group, while the majority of deaths were centered in the 1 to 4-year-old age group. Thus, child burns warrant attention. Almost 90% of burn deaths worldwide occur in low- and middle-income countries. China, the world largest developing nation, faces the issue of burns. It has the most documented cases, accounting for 12% of newly discovered cases worldwide.^[1]^ Since 1978, numerous articles on burns have been published in domestic and international journals. However, there are few studies addressing burn encephalopathy and the application of extracorporeal membrane oxygenation (ECMO).

Burns are typically associated with immunological dysfunction, infectious inflammation, hypermetabolic changes, and distributed shock, which may result in multiple organ failures. Burns can induce a common systemic inflammatory response that can disrupt the blood-brain barrier, resulting in neuronal damage and life-threatening brain edema. In addition, inhalation of smoke or chemical combustion products associated with burns, such as carbon monoxide, hydrogen cyanide, and other toxic components, can cause inhalation injury, exacerbate lung function damage, and cerebral hypoxia, leading to immediate and delayed neurological damage. Various neurological complications caused by smoke inhalation are among the most severe burn-related injuries. Thus, the brain is one of the organs that is severely damaged in burn victims and has been proven to be highly correlated with death. Research has shown that hypoxic brain injury is the main cause of age-dependent mortality in up to 10% of severely burned patients.^[2]^ Brain damage caused by burns is particularly severe in children with incomplete neurological development,^[3]^ and it is also one of the leading causes of death in pediatric burn patients.^[4,5]^ Maintaining adequate oxygenation and managing inhalation injuries are critical components of the treatment for burn encephalopathy. ECMO is an effective and feasible method for treating hypoxemia and cardiac failure, but additional research is needed to determine the safety, feasibility, and usefulness of ECMO in patients with burn encephalopathy.^[6]^ Here, we describe a case of inhalation injury resulting from burns, analyze the treatment and related examination results, and review the relevant literature. Our findings may provide insights for ECMO treatment in children with burns and encephalopathy.

## 2. Case presentation

The patient is a 7-year-old boy who was admitted to the hospital with “loss of consciousness due to smoke suffocation for more than 1 day.” One day ago, the boy lost consciousness after inhaling a large amount of smoke at the fire site. After continuous cardiopulmonary resuscitation, he recovered his autonomous heart rate, but not his autonomous consciousness and breathing. Despite tracheal intubation with a ventilator and a large dose of vasoactive medications, the boy continued to exhibit signs of severe hypoxemia and arrhythmia. Thereafter, with the use of fiberoptic bronchoscopy, the blood oxygen saturation improved in comparison to the previous treatment. However, the child heart rate and blood pressure are still unstable, and drug control is ineffective. In this incident, the child grandmother and sister were killed. The boy was previously healthy and has no pulmonary or craniocerebral diseases.

Physical examination: Total body surface area burn is 0%, T 31.9°C, P 104 beats/minute, respiration rate 15 breaths/minute, blood pressure 123/105 mm Hg, ECMO circulation support, ventilator application, deep coma, Glasgow Coma Score of 3 points, bilateral pupil size, no light reflex extraction, bilateral lung auscultation breathing sounds thick, no obvious dry and wet rales, arrhythmia, low and blunt heart sounds, capillary filling time of 4 seconds, abdominal wall reflex extraction, testicular reflex extraction.

**Figure 1.**
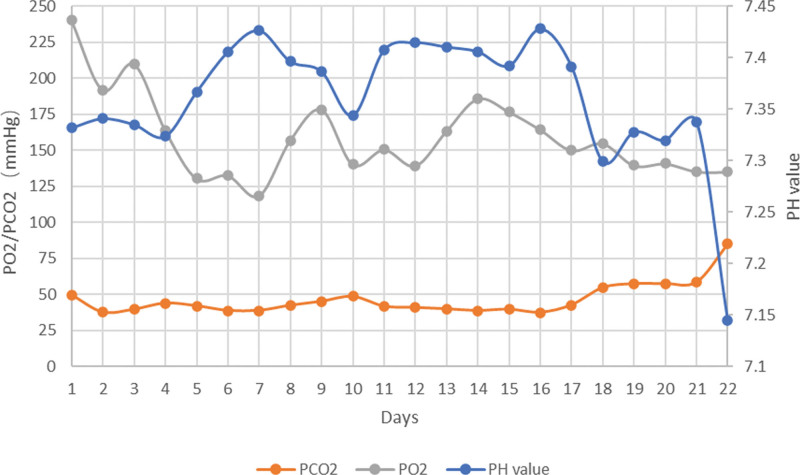
Changes in blood partial pressure of carbon dioxide, oxygen, and pH value during the course of disease.

**Figure 2.**
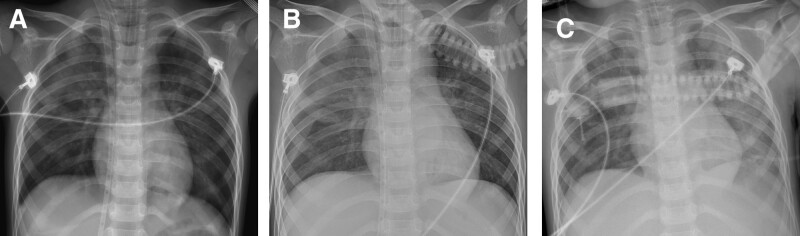
X-ray: The first day of the course of disease: pulmonary edema (A); The 4th d: pulmonary edema are slightly improved compared to the previous film, with the possibility of incomplete expansion of the right upper lung and pleural effusion on the right side (B); The 16th d: the lesions in both lungs are more severe than on the previous film, and there may be fluid accumulation in the left pleural cavity (C).

**Figure 3.**
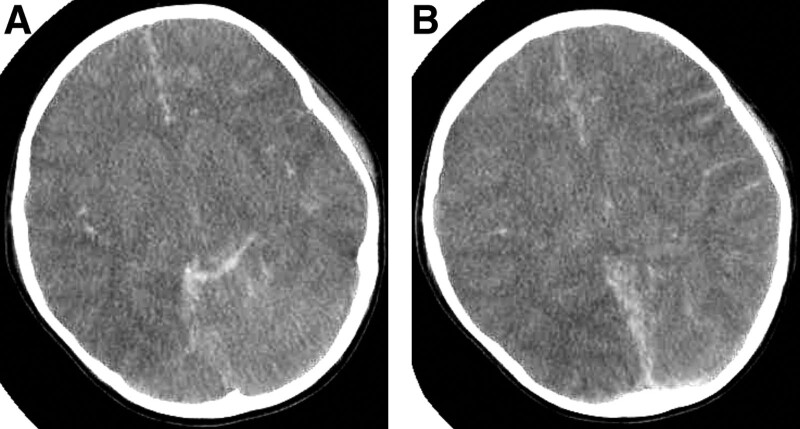
Head CT: The midline structure of the brain is centered, the whole brain parenchyma is swollen, the supratentorial ventricular compression disappears, the density of the brain parenchyma is low, the gray white matter boundary is unclear, and the midline, bilateral cerebral sulci, and tentorial margin of the cerebellum have linear density increases, and the medullary structure is not clearly displayed. No obvious bone abnormality is found in the skull. The whole brain swelling may change with a small amount of subarachnoid hemorrhage, and cerebral hernia may occur (A, B). CT = computed tomography.

In the later stages of the disease, auxiliary examination and blood gas analyses suggest acidosis and carbon dioxide retention (Fig. 1). The results of a routine blood examination revealed an increase in white blood cells and hypersensitive C-reactive protein. Fiberoptic bronchoscopy revealed a large amount of black carbon-like aspirates from the trachea. The echocardiographic results of the child heart revealed a widespread decrease in global cardiac activity and poor cardiac function at admission (ejection fraction: 0.3). After ECMO application, echocardiographic examination showed that the cardiac function was significantly improved, with an left ventricular ejection fraction of 56%. X-ray examination of the lungs revealed signs of pulmonary edema, pulmonary insufficiency, and pleural effusion (Fig. 2). The computed tomography scan of the head indicated changes in the overall brain edema, accompanied by a small amount of subarachnoid hemorrhage and cerebral hernia (Fig. 3). During electroencephalogram (EEG) recording, the amplitude of brain waves is low and there is no change, and a small number of others can be seen δθ. The activity was sporadic or transient, and stimulation of the boy during recording did not result in brain wave changes. EEG impression: EEG activity is relatively depressed and lacks change. The last EEG results indicate continuous electrical rest, indicating brain death.

Treatment and prognosis: The boy was diagnosed with an inhalation injury, burn encephalopathy, and tracheal intubation and ventilation were administered. Despite fiberoptic bronchoscopy-assisted bedside therapy, arrhythmia persists. On the second day of the disease progression, ECMO treatment was performed, and the child arrhythmia improved and blood circulation gradually stabilized. After removing ECMO and switching to synchronized intermittent mechanical ventilation mode for assisted breathing, the boy was unable to resume autonomic respiration, as evidenced by pupil dilation. The family was advised that the child prognosis was bad due to the presence of a central nervous system injury, and the parent consented to discontinuing treatment. The patient was extubated, disconnected from the monitor, discontinued tube feeding, and all infusion therapy was discontinued. At this point, only palliative and compassionate care was provided. The patient ultimately had respiratory and cardiac arrest due to irreparable burn encephalopathy resulting from an inhalation injury and died 22 days after admission.

## 3. Literature review and discussion

The incidence of multiple organ dysfunction in severely burned patients has increased significantly. Burn encephalopathy is a neurological injury caused by burns and can occur in up to 80% of burn and inhalation injury patients.^[7]^ Notably, intracranial injury secondary to burn-related trauma or hypoxic brain injury may damage and delay recovery. Initial brain damage may be caused by hypoxia and the neurotoxicity of inhaling smoke. Inhalation of smoke can cause dilation and congestion of cerebral blood vessels, significantly reducing the number of normal blood vessels, loss of integrity of the vascular basement membrane, rupture of cerebral blood vessels, damage to the blood-brain barrier, and disrupt the energy metabolism of the brain, leading to profound damage to the brain after severe burns.^[8]^ Changes in the tissue morphology and metabolic function of the brain after burns,^[9]^ can lead to psychosocial disorders, cognitive behavioral abnormalities, and death.

Consequently, clinical treatment of burn victims must prioritize the protection of both the lungs and the brain. Pediatric burn patients should be considered for ECMO treatment when maximal medical treatment fails to improve their clinical state. ECMO is an enhanced extracorporeal circulation therapeutic approach. It is an advanced form of extracorporeal life support that can be used as a heart and artificial membrane lung to provide gas exchange and systemic perfusion when the heart and lungs cannot fully function.^[10]^ These indications can be divided into several categories, such as hypoxic respiratory failure, hypercapnia respiratory failure, cardiogenic shock, and cardiac arrest. Therefore, ECMO can provide time for the organ function to recover following inhalation injury or other organ dysfunction associated with burns, thereby improving the survival rate of burn patients.^[11]^ Due to the constant growth of indications and technology, ECMO has become increasingly prevalent in clinical applications. Its use in children with burns has gradually increased in recent years. ECMO can save the lives of burn patients, but it carries a high risk, and its adverse results may be caused by brain injury.^[12,13]^ Indeed, nerve injury increased dramatically in ECMO patients. Studies have demonstrated that 15% to 35% of ECMO patients developed neurological damage.^[14]^ Due to the use of potent sedatives, anticoagulants, and the closure of the jugular artery and vein during ECMO, it is an invasive and abnormal physiological state that may cause brain damage. In addition, bleeding and thrombosis are still major consequences of ECMO, with intubation sites and the brain being potential bleeding sites. Moreover, massive cerebral hemorrhage is the most terrifying consequence of ECMO therapy. Brain cell edema, vasospasm, and accompanying clinical symptoms induced by increased intracranial pressure, such as headache, dizziness, nausea, vomiting, hemiplegia, and behavioral and cognitive abnormalities, may be the pathological mechanism of ECMO brain injury. The primary factor that determines the outcome of ECMO is brain damage. However, current research on the effect of ECMO on the prognosis of children with burn encephalopathy is limited. In this study, there was no evidence of ECMO-related brain damage, and the child cardiovascular, respiratory, and circulatory systems gradually recovered, his life weight stabilized, and he was able to successfully withdraw from ECMO support treatment. However, the child eventually died due to severe damage to the central nervous system and blood coagulation system caused by early burns.^[15]^ Therefore, it is essential to closely monitor neural function,^[14]^ manage anticoagulant therapy, and prevent clot formation in the ECMO circuit,^[16]^ in children receiving ECMO. Future research should focus on determining the optimal ECMO treatment strategy for patients with burns and brain injuries. Monitoring brain function and blood coagulation is an important aspect of the management of patients undergoing ECMO treatment. Physicians should establish noninvasive neurological monitoring during ECMO, including EEG, somatosensory evoked potential, and transcranial Doppler, Imaging examination, as well as real-time monitoring of blood coagulation function, to detect brain injury early, actively treat, and improve prognosis.^[17,18]^

In addition, previous studies have shown that not all patients are treated with ECMO to improve their condition. Before applying ECMO to burn children, a comprehensive evaluation of the child condition is required. ECMO support may need to be discontinued if certain diseases are irreversible, unresponsive to treatment, or may lead to progressive sexual organ dysfunction, such as liver failure or severe neurological damage.^[19]^ According to the ECMO absolute contraindication guidelines established by extracorporeal life support organization, all of these are considered fatal and irreversible diseases. These include fatal chromosomal abnormalities (trisomy 13 and 18), severe neurological damage ("intracranial hemorrhage with space-occupying effects”), incurable malignant tumors, and lung disease in recipients of allogeneic bone marrow transplantation.^[20]^

Notably, in addition to the use of ECMO to improve cerebral blood supply and oxygen supply, there are currently several studies that provide adjunctive treatment options for patients with burns and brain injuries. Studies have found that the levels of various circulating proinflammatory cytokines in burn patients correlate with the severity and outcome of disease damage. Estrogen can decrease proinflammatory cytokine levels and protect the brain from burns. The protective effect of estrogen may reduce early brain inflammation and apoptotic cell death by elevating the level of phosphorylated Akt and activating ERK.^[21]^ Gelsolin is an important actin-binding protein that can improve encephalitis and apoptosis by accelerating monocyte recruitment and downregulating phosphorylated ERK1/expression, as well as reducing mortality by improving peripheral T-cell function.^[22]^ Mat is a quinoline alkaloid isolated from Sophora flavescens that can play an anti-anxiety and anti-depression role by inhibiting JNK-mediated apoptosis/inflammatory signal transduction, oxidative stress, and reversing the burn-induced downregulation of BDNF/VEGF in the hippocampus.^[23]^ After burns, nNOS in the frontal cortex, striatum, and midbrain are associated with acute inflammatory responses. Captopril treatment eliminates the nNOS response in the frontal cortex and midbrain.^[24]^ Early single-dose pentoxifylline reduces brain inflammation and apoptosis up to 16 hours after injury.^[25]^ Overall, ECMO is an effective life-saving treatment for children with burns and respiratory and circulatory failure when routine treatment cannot improve their condition.

## 4. Conclusion

Currently, only a few published reports describe burn encephalopathy. In this case report, the patient condition worsens despite receiving conventional, second-line, and third-line regimens. ECMO is initiated due to malignant arrhythmias and hypoxemia. The boy condition improved as a result of ECMO treatment, and he was eventually able to withdraw from ECMO, but he died due to burns and encephalopathy. Due to the lack of specific clinical symptoms in patients with burn encephalopathy, timely diagnosis and treatment are often challenging. In the majority of patients with burns and brain injuries, consciousness disturbance is the initial clinical manifestation. As the disease progresses, additional symptoms such as convulsions, low amplitude of EEG, and lack of response to stimuli may manifest. Therefore, the possibility of burn encephalopathy should be considered for burn patients with consciousness disorders, and their brain function should be assessed as soon as possible. In cases where there is a high clinical suspicion of brain injury, treatment should be initiated as soon as possible, even while awaiting test results. ECMO is still an effective treatment for such patients.

## Acknowledgments

The authors would like to thank Dr Win Topatana from Zhejiang University for his assistance during the revision of this paper.

## Author contributions

**Conceptualization:** Yanfei Wang, Linhua Tan.

**Data curation:** Yanfei Wang, Kelei Deng, Linhua Tan.

**Formal analysis:** Yanfei Wang.

**Funding acquisition:** Linhua Tan.

**Resources:** Junjie Qian, Linhua Tan.

**Supervision:** Linhua Tan.

**Writing – original draft:** Yanfei Wang, Kelei Deng.

**Writing – review & editing:** Yanfei Wang, Junjie Qian.
